# Efficiency Fluctuations in a Quantum Battery Charged by a Repeated Interaction Process

**DOI:** 10.3390/e24060820

**Published:** 2022-06-13

**Authors:** Felipe Barra

**Affiliations:** Departamento de Física, Facultad de Ciencias Físicas y Matemáticas, Universidad de Chile, Santiago 8370415, Chile; fbarra@dfi.uchile.cl

**Keywords:** quantum collision models, ergotropy, quantum batteries, efficiency fluctuations

## Abstract

A repeated interaction process assisted by auxiliary thermal systems charges a quantum battery. The charging energy is supplied by switching on and off the interaction between the battery and the thermal systems. The charged state is an equilibrium state for the repeated interaction process, and the ergotropy characterizes its charge. The working cycle consists in extracting the ergotropy and charging the battery again. We discuss the fluctuating efficiency of the process, among other fluctuating properties. These fluctuations are dominated by the equilibrium distribution and depend weakly on other process properties.

## 1. Introduction

Repeated interaction schemes, also known as collisional models [1,2,3,4,5,6], have played a vital role in the development of quantum optics [7,8,9,10] and the rapid evolution of quantum thermodynamics [11,12,13,14,15]. The idealized and straightforward formalism has been crucial to designing and understanding quantum devices such as information engines [16,17,18,19], heat engines [12,20,21,22,23], and quantum batteries [24,25,26,27,28,29,30,31,32,33,34]. Recently, it was realized that the framework can be extended to deal with macroscopic reservoirs [23,35], expanding the reach of applications in quantum thermodynamics. For comprehensive reviews of the method and its applications, see [36,37].

In the simplest scenario, many copies of an auxiliary system in the Gibbs equilibrium thermal state interact sequentially with a system of interest. Each interaction step is described by a completely positive trace-preserving (CPTP) map [38]. The repeated interaction process corresponds to concatenations of the map, which eventually will bring the system to a nonequilibrium steady state or an equilibrium state. In equilibrium, heat does not flow to the environment, and entropy is not produced. When the repeated interaction brings the system to an equilibrium state, we say that we iterate a map with equilibrium. In this paper, we apply this framework to study a quantum battery.

Quantum technologies, such as quantum computing, communication, and sensing, are supported by the quantum storage and transfer of energy. Implementing fast and reliable quantum batteries in these technologies may improve their functionality. Different quantum batteries have been proposed to achieve these goals [39,40,41,42]. One paradigmatic setup considers the battery to be composed of noninteracting qubits. Global operations, such as charging or discharging the battery by coupling all qubits to a single optical cavity mode, boost its performance in power [28,29,30,31] and reliability [43].

The most straightforward repeated interaction model for a quantum battery considers nonequilibrium auxiliary systems supplying the energy. However, the process of sustaining the charged state is dissipative. Reference [26] proposed a different kind of quantum battery where the charged state corresponds to the equilibrium state of the process. The work in the recharging stage provides the energy, which is preserved without dissipation in the equilibrium state as long as the battery–environment interaction remains under control. In actual physical implementations, other exchanges can still cause energy leakage. The battery’s charge is characterized by its ergotropy [44], i.e., the maximum amount of energy extracted with a unitary process. Once removed, a repeated interaction process recharges the battery. In this way, we have a thermodynamic cycle.

The recharging energy and the ergotropy delivered by the quantum battery are averaged values that are relevant for several cycles or many batteries working parallel. In a single cycle, one can observe fluctuations when observing these energies. Therefore, their study is relevant for the reliability of the device. The two-point measurement scheme [45] is appropriate for describing these thermal and quantum fluctuations that reveal essential properties of the process [46,47,48,49]. Other sources of randomness in the operation of a battery can arise from changes in the evolution operator [50,51,52], Hamiltonian [53], and initial condition [54]. We do not take them into account. Closer to the spirit of this work are studies of work fluctuations in the charging or discharging process of isolated quantum batteries [55,56,57].

Thus, in this work, we take the dissipative quantum battery [26] and study fluctuations in the thermodynamic quantities such as heat and work during the charging phase and the efficiency fluctuations of the cycle. Efficiency fluctuations are significant in assessing the performance of a machine. They have drawn recent attention in classical [58,59,60,61,62,63,64,65,66,67,68] and quantum [21] engines. Evaluating the fluctuations requires detailed information about the bath and the process [45]. However, a key simplification arises because we deal with maps with equilibrium, allowing us to determine the statistics of the fluctuations. We will illustrate this using two examples.

For completeness, we also consider equilibrium fluctuations. We evaluate the probability of performing work or absorbing heat while keeping the (average) charge in the battery. We compare our findings with the equilibrium fluctuations in a process with a Gibbs equilibrium state.

The remainder of this article is organized as follows. In Section 2, we review the thermodynamics for CPTP maps, emphasizing the results for maps with equilibrium. Then, in Section 3, we introduce our system of study, namely the equilibrium quantum battery proposed in [26]. Section 4 discusses the stochastic versions of the thermodynamic equalities and laws, emphasizing the results for maps with equilibrium again. Subsequently, in Section 5, we evaluate these fluctuations in two illustrative examples. We conclude this article in Section 6.

## 2. Thermodynamic Description for Completely Positive Trace-Preserving Maps

Consider a system *S* and a system *A* that jointly evolve under the unitary $U=e^{-i\frac{\tau }{\hbar }\left(H_{S}+H_{A}+V\right)}$. The Hamiltonians $H_{S}$ and $H_{A}$ of *S* and *A*, respectively, are constant in time. The coupling between *S* and *A* during the time interval (0,τ) is given by the interaction energy *V* and vanishes for t<0 and t>τ.

Initially, *S* and *A* are uncorrelated; i.e., their density matrix is the tensor product of the respective density matrices $\rho_{\mathrm{tot}}=\rho_{S}⊗\omega_{\beta }\left(H_{A}\right)$, where $\omega_{\beta }\left(H_{A}\right)=\frac{e^{-\beta H_{A}}}{Z_{A}}$ is the Gibbs thermal state for *A* with β as the inverse temperature, and $Z_{A}=\mathrm{Tr}\;e^{-\beta H_{A}}$. After the lapse of time τ, the initial state $\rho_{\mathrm{tot}}$ changes to a new state,

(1) $$\rho_{\mathrm{tot}}^{\prime }=U\left(\rho_{S}⊗\omega_{\beta }\left(H_{A}\right)\right)U^{†}.$$

In the following, we denote $\rho_{S}^{\prime }={\mathrm{Tr}}_{A}\rho_{\mathrm{tot}}^{\prime }$ and $\rho_{A}^{\prime }={\mathrm{Tr}}_{S}\rho_{\mathrm{tot}}^{\prime }$, where ${\mathrm{Tr}}_{X}$ is the partial trace over subsystem *X*. By tracing out *A*, one obtains a CPTP map E for the system *S* evolution

(2) $$\rho_{S}^{\prime }=E\left(\rho_{S}\right)={\mathrm{Tr}}_{A}\left[U\left(\rho_{S}⊗\omega_{\beta }\left(H_{A}\right)\right)U^{†}\right].$$

The energy change of *S*

(3) $$\Delta E=\mathrm{Tr}[H_{S}\left(\rho_{S}^{\prime }-\rho_{S}\right)],$$

can be written as the sum of

(4) $$Q=\mathrm{Tr}[H_{A}\left(\omega_{\beta }\left(H_{A}\right)-\rho_{A}^{\prime }\right)],$$

and

(5) $$W=\mathrm{Tr}[\left(H_{S}+H_{A}\right)\left(\rho_{\mathrm{tot}}^{\prime }-\rho_{\mathrm{tot}}\right)],$$

satisfying the first law ΔE=W+Q. Note that −Q is the energy change of *A*, we call *Q* the heat, and *W* is the energy change of the full S+A system, which we call the switching work because it is due to the energy cost of turning on and off the interaction *V* at the beginning and end of the process, respectively [69,70].

Consider the von Neumann entropy change

(6) $$\Delta S_{\mathrm{vN}}=-\mathrm{Tr}\left[\rho_{S}^{\prime }\ln\rho_{S}^{\prime }\right]+\mathrm{Tr}\left[\rho_{S}\ln\rho_{S}\right]$$

of system *S* and the heat *Q* given in Equation (4). The entropy production, $\Sigma =\Delta S_{\mathrm{vN}}-\beta Q$, is also given by [71]

(7) $$\Sigma =D(\rho_{\mathrm{tot}}^{\prime }||\rho_{S}^{\prime }⊗\omega_{\beta }\left(H_{A}\right))\geq 0,$$

with D(a||b)≡Tr[alna]−Tr[alnb]. The inequality in Equation (7) corresponds to the second law. Note that auxiliary system *A* does not need to be macroscopic; nevertheless, we will call it the bath.

As in standard thermodynamics, analyzing the process $\rho_{S}\to \rho_{S}^{\prime }=E\left(\rho_{S}\right)$, in terms of ΔE=W+Q and $\Sigma =\Delta S_{\mathrm{vN}}-\beta Q\geq 0$ with the quantities given in Equations (3)–(7) is very useful. Note that for their evaluation, particularly for the work, Equation (5), and entropy production, Equation (7), we need to know the full state $\rho_{\mathrm{tot}}^{\prime }$.

### Maps with Thermodynamic Equilibrium

In a repeated interaction process, one concatenates *L* CPTP maps $E^{L}\equiv E\circ \cdots \circ E\left(\cdot \right)$ to describe a sequence of evolutions of a system coupled to an auxiliary thermal system for a given lapse of time τ. With each map E, a new fresh bath is introduced that exchanges heat with the system during the time that the interaction is turned on. The concatenated map $E^{L}$ is also a CPTP map. The total work performed is the sum of the work performed by switching on and off the interaction energy with each bath. Similarly, the total heat is the sum of the heat exchanged with each bath.

Let us assume that the map E has an attractive invariant state $\bar{\rho }$, defined as

$$\lim_{L\to \infty }E^{L}\left(\rho_{S}\right)=\bar{\rho },\;\forall \rho_{S},$$

and $\bar{\rho }=E\left(\bar{\rho }\right)$. The process $\bar{\rho }\to E\left(\bar{\rho }\right)$ is thermodynamically characterized by $\Delta S_{\mathrm{vN}}=0=\Delta E$; see Equations (3) and (6). If the entropy produced by the action of the map E on $\bar{\rho }$ is Σ>0, then we say that the invariant state is a nonequilibrium steady state. The invariant state is an *equilibrium state* if Σ=0, i.e., if the entropy production, Equation (7), vanishes by the action of E on $\bar{\rho }$. Maps with these particular states are called maps with equilibrium [72,73].

According to Equation (7), Σ=0 for the steady state $\bar{\rho }$ if and only if $\bar{\rho }⊗\omega_{\beta }\left(H_{A}\right)=U\left(\bar{\rho }⊗\omega_{\beta }\left(H_{A}\right)\right)U^{†}$. Equivalently, if the unitary *U* in Equation (1) satisfies $[U,H_{0}+H_{A}]=0$, where $H_{0}$ is an operator in the Hilbert space of the system, then the product state $\omega_{\beta }\left(H_{0}\right)⊗\omega_{\beta }\left(H_{A}\right)$, with $\omega_{\beta }\left(H_{0}\right)=\frac{e^{-\beta H_{0}}}{Z_{0}}$, where $Z_{0}=\mathrm{Tr}\left[e^{-\beta H_{0}}\right]$, is invariant under the unitary evolution in Equation (1) and $\bar{\rho }=\omega_{\beta }\left(H_{0}\right)$ is an equilibrium state for the map in Equation (2).

It follows from $[U,H_{0}+H_{A}]=0$ that the heat, Equation (4), and work, Equation (5), simplify to

(8) $$Q=\mathrm{Tr}[H_{0}\left(\rho_{S}^{\prime }-\rho_{S}\right)]$$

and

(9) $$W={\mathrm{Tr}}_{S}\left[\left(H_{S}-H_{0}\right)\left(\rho_{S}^{\prime }-\rho_{S}\right)\right].$$

The entropy production also reduces to an expression that does not involve the state of the bath. Indeed, we obtain

(10) $$\Sigma =D(\rho_{S}\left|\right|\omega_{\beta }\left(H_{0}\right))-D(\rho_{S}^{\prime }\left|\right|\omega_{\beta }\left(H_{0}\right)),$$

which is positive due to the contracting character of the map [38]. The averaged thermodynamic quantities for a map with equilibrium are only determined by the properties of the system of interest.

If $H_{0}=H_{S}$, then the map is called thermal [74,75]. The equilibrium state is the Gibbs state $\omega_{\beta }\left(H_{S}\right)=e^{-\beta H_{S}}/Z_{S}$ with $Z_{S}=\mathrm{Tr}\left[e^{-\beta H_{S}}\right]$, and the agent is passive because W=0 for every initial state $\rho_{S}$; see Equation (9).

When $H_{0}\neq H_{S}$, an active external agent has to provide (or extract) work to perform the map on a state $\rho_{S}$. However, once the system reaches the equilibrium state $\omega_{\beta }\left(H_{0}\right)$, the process $\omega_{\beta }\left(H_{0}\right)\to E\left(\omega_{\beta }\left(H_{0}\right)\right)=\omega_{\beta }\left(H_{0}\right)$ is performed with W=0; see Equation (9), and Σ=0.

Let us end this section with the following remark. Since the total evolution operator $U=e^{-i\frac{\tau }{\hbar }\left(H_{S}+H_{A}+V\right)}$ is time-independent, the equilibrium condition is satisfied by finding $H_{0}$ and *V* such that $[H_{0},H_{S}]=0$ and $[H_{0}+H_{A},V]=0$ [26]. In this case, $H_{S}$ and $H_{0}$ share the same eigenbasis. To simplify the discussion of fluctuations, we consider non-degenerate eigenenergies. We denote the eigensystems as

$$H_{S}|n\rangle =E_{n}|n\rangle ,\;H_{0}|n\rangle =E_{n}^{0}|n\rangle .$$

with increasing order $E_{1}<E_{2}<\cdots <E_{N}$ for the eigenenergies. The eigenvalues $E_{n}^{0}$ are not necessarily ordered, but there is always a permutation that we call π of $\left(1,\ldots ,N\right)\to \left(\pi_{1},\ldots ,\pi_{N}\right)$ such that $E_{\pi_{1}}^{0}\leq \cdots \leq E_{\pi_{N}}^{0}$.

## 3. The Battery

As is well known, the Gibbs state $\omega_{\beta }\left(H_{S}\right)$ is passive; i.e., one cannot decrease (extract) its energy with a unitary operation [76,77]. This is not true for the equilibrium state

(11) $$\omega_{\beta }\left(H_{0}\right)=\sum_{n}\frac{e^{-\beta E_{n}^{0}}}{Z_{0}}|n\rangle \langle n|,$$

if a pair (j,k) exists such that $\left(E_{j}-E_{k}\right)\left(E_{j}^{0}-E_{k}^{0}\right)<0$. In that case, the unitary operator *u* with matrix elements $u_{ij}=\langle i|u|j\rangle =\delta_{\pi_{i},j}$ extracts the ergotropy [44]

(12) $$W\left[\omega_{\beta }\left(H_{0}\right)\right]=\sum_{n=1}^{N}\left(E_{\pi_{n}}-E_{n}\right)\frac{e^{-\beta E_{\pi_{n}}^{0}}}{Z_{0}}>0,$$

where π is the permutation that orders $E_{n}^{0}$ increasingly.

Once the ergotropy is extracted, the system is left in the passive state

(13) $$\sigma_{\omega_{\beta }\left(H_{0}\right)}=u\omega_{\beta }\left(H_{0}\right)u^{†}=\sum_{n=1}^{N}\frac{e^{-\beta E_{\pi_{n}}^{0}}}{Z_{0}}|n\rangle \langle n|.$$

An equilibrium quantum battery was proposed in [26] based on that observation. The system is driven by a repeated interaction process described by a map E with equilibrium $\omega_{\beta }\left(H_{0}\right)$. Once the equilibrium is reached, it is kept with no cost (W=0), energy does not leak from it, and the battery’s charge, characterized by the ergotropy $W[\omega_{\beta }\left(H_{0}\right)]$, is preserved. Equilibrium states with ergotropy are called active.

The thermodynamic cycle is as follows: The battery starts in the active equilibrium state, and then the ergotropy (12) is extracted, leaving the battery in the passive state (13) from which the repeated interaction process $\lim_{L\to \infty }E^{L}\left(\sigma_{\omega_{\beta }\left(H_{0}\right)}\right)$ recharges it. As a consequence of the second law, the recharging work $W_{R}={\mathrm{Tr}}_{S}\left[\left(H_{S}-H_{0}\right)\left(\omega_{\beta }\left(H_{0}\right)-\sigma_{\omega_{\beta }\left(H_{0}\right)}\right)\right]$ is never smaller that the extracted ergotropy. In this way, the thermodynamic efficiency

(14) $$\begin{array}{c} 0\leq \eta_{\mathrm{th}}\equiv \frac{W[\omega_{\beta }\left(H_{0}\right)]}{W_{R}}\leq 1, \end{array}$$

which is the ratio of the wanted resource over the invested, characterizes the operation of the device.

## 4. Fluctuations

### 4.1. Repeated Interaction for a Map with Equilibrium

The thermodynamic quantities in Equations (3)–(7) were obtained as the average over their stochastic versions defined over trajectories using a two-point measurement scheme in [72]. Since all interesting density matrices $\omega_{\beta }\left(H_{S}\right),\omega_{\beta }\left(H_{0}\right)$, and $\sigma_{\omega_{\beta }\left(H_{0}\right)}$ are diagonal in the system energy basis, we need only projective energy measurement in this work.

A trajectory $\gamma =\{n;i_{1},j_{1},\ldots ,i_{L},j_{L};m\}$ for the recharging process is defined by the initial and final, $\varepsilon_{i_{k}}$ and $\varepsilon_{j_{k}}$, energy results for each auxiliary thermal system and $E_{n}$ and $E_{m}$ for the system. According to the two-point measurement scheme [45], its probability is

(15) $$P_{\gamma }^{\left(L\right)}=|\langle j_{1}\cdots j_{L}m|U_{L}\cdots U_{1}|i_{1}\cdots i_{L}n{\rangle |}^{2}\frac{e^{-\beta \sum_{k=1}^{L}\varepsilon_{i_{k}}}}{Z_{A}^{L}}p_{\mathrm{ini}}\left(n\right),$$

where $p_{\mathrm{ini}}\left(n\right)$ is the probability that the initial state of the system is |n〉; see Appendix A. We now associate the stochastic thermodynamic quantities with these trajectories. The stochastic heat flow to the system $q_{\gamma }$ corresponds to the negative energy change of the bath, i.e., $q_{\gamma }=\sum_{k=1}^{L}\left(\varepsilon_{i_{k}}-\varepsilon_{j_{k}}\right)$. According to the first law of stochastic thermodynamics [47], the stochastic work is given by

(16) $$w_{\gamma }=\Delta e_{\gamma }-q_{\gamma },$$

where $\Delta e_{\gamma }=E_{m}-E_{n}$ is the stochastic energy change. These fluctuating quantities are studied through their distributions

(17) $$p_{w}^{\left(L\right)}\left(x\right)=\sum_{\gamma }\delta \left(x-w_{\gamma }\right)P_{\gamma }^{\left(L\right)},\;p_{\Delta e}^{\left(L\right)}\left(x\right)=\sum_{\gamma }\delta \left(x-\Delta e_{\gamma }\right)P_{\gamma }^{\left(L\right)},\;p_{q}^{\left(L\right)}\left(x\right)=\sum_{\gamma }\delta \left(x-q_{\gamma }\right)P_{\gamma }^{\left(L\right)},$$

and, as for the averaged thermodynamic quantities, we need information on the state of the whole system to evaluate them. However, for maps with equilibrium, a stochastic trajectory is determined by the pair γ={n,m}; see Appendix A. Consequently these formulas simplify and become, $q_{\gamma }=E_{m}^{0}-E_{n}^{0},w_{\gamma }=E_{m}-E_{m}^{0}-\left(E_{n}-E_{n}^{0}\right)$ with the distributions

(18) $$p_{\Delta e}^{\left(L\right)}\left(x\right)=\sum_{n,m}\delta \left(x-\left[E_{m}-E_{n}\right]\right)P_{n\to m}^{\left(L\right)},$$

(19) $$p_{w}^{\left(L\right)}\left(x\right)=\sum_{n,m}\delta \left(x-\left[\left(E_{m}-E_{m}^{0}\right)-\left(E_{n}-E_{n}^{0}\right)\right]\right)P_{n\to m}^{\left(L\right)},$$

(20) $$p_{q}^{\left(L\right)}\left(x\right)=\sum_{n,m}\delta \left(x-\left[E_{m}^{0}-E_{n}^{0}\right]\right)P_{n\to m}^{\left(L\right)},$$

and the trajectory probability

(21) $$P_{n\to m}^{\left(L\right)}=\langle m|E^{L}\left(|n\rangle \langle n|\right)|m\rangle p_{\mathrm{ini}}\left(n\right)={\left(T^{L}\right)}_{m|n}\;p_{\mathrm{ini}}\left(n\right),$$

in terms of the initial probability $p_{\mathrm{ini}}\left(n\right)$ and of the *L* power of the stochastic matrix $T_{m|n}=\langle m|E\left(|n\rangle \langle n|\right)|m\rangle$.

The averages $\int xp_{\Delta e}^{\left(L\right)}\left(x\right)dx,\int xp_{w}^{\left(L\right)}\left(x\right)dx,\int xp_{q}^{\left(L\right)}\left(x\right)dx$ reproduce Equations (3), (8) and (9) with $\rho_{S}^{\prime }=E^{L}\left(\rho_{S}\right)$ and $\rho_{S}=\sum_{n}p_{\mathrm{ini}}\left(n\right)|n\rangle \langle n|$.

### 4.2. Fluctuations in the Equilibrium State

As noted before, all averaged thermodynamic quantities ΔE=ΔS=Σ=W=Q=0 vanish for a process in equilibrium. So, on average, the process $\omega_{\beta }\left(H_{0}\right)\to E\left(\omega_{\beta }\left(H_{0}\right)\right)=\omega_{\beta }\left(H_{0}\right)$ has no energy cost. However, if $H_{0}\neq H_{S}$, the agent is still active due to non-vanishing work fluctuations. For thermal maps, $H_{0}=H_{S}$ and Equation (19) gives $p_{w}^{\left(L\right)}\left(x\right)=\delta \left(x\right)$. The external agent is truly passive.

To analyze equilibrium fluctuations, we use Equations (18)–(20) with $p_{\mathrm{ini}}\left(n\right)=\frac{e^{-\beta E_{n}^{0}}}{Z_{0}}$.

### 4.3. Recharging Process

Since the recharging process starts from $\sigma_{\omega_{\beta }\left(H_{0}\right)}$, we take $p_{\mathrm{ini}}\left(n\right)=e^{-\beta E_{\pi_{n}}^{0}}/Z_{0}$—see Equation (13)—in the distribution Equations (18)–(20).

Since the charged state $\omega_{\beta }\left(H_{0}\right)$ is reached asymptotically, we take L→∞ to charge the battery fully.

Moreover, since E has a unique equilibrium state, we will find that *T* is a regular stochastic matrix [78], implying that $\lim_{L\to \infty }{\left(T^{L}\right)}_{m|n}=e^{-\beta E_{m}^{0}}/Z_{0},\forall n$. Therefore, the limit in Equation (21)

(22) $$P_{n\to m}^{\left(\infty \right)}=p_{\mathrm{ini}}\left(n\right)e^{-\beta E_{m}^{0}}/Z_{0}=e^{-\beta (E_{\pi_{n}}^{0}+E_{m}^{0})}/Z_{0}^{2},$$

is independent of the map’s details. Interestingly, the rate of convergence of $T^{L}$ to the equilibrium distribution depends on the map E parameters. We later discuss the fluctuations of a concatenated process $E^{L}$ with finite *L*.

The average of the stochastic energy change in the recharging process

(23) $${\langle \Delta e_{\gamma }\rangle }^{\left(\infty \right)}\equiv \sum_{n,m}\left(E_{m}-E_{n}\right)P_{n\to m}^{\left(\infty \right)}=\mathrm{Tr}\left[H_{S}\left(\omega_{\beta }\left(H_{0}\right)-\sigma_{\omega_{\beta }\left(H_{0}\right)}\right)\right]=W\left(\omega_{\beta }\left(H_{0}\right)\right)$$

is the ergotropy. The average stochastic work

(24) $${\langle w_{\gamma }\rangle }^{\left(\infty \right)}\equiv \sum_{n,m}\left(\left(E_{m}-E_{m}^{0}\right)-\left(E_{n}-E_{n}^{0}\right)\right)P_{n\to m}^{\left(\infty \right)}=\mathrm{Tr}\left[\left(H_{S}-H_{0}\right)\left(\omega_{\beta }\left(H_{0}\right)-\sigma_{\omega_{\beta }\left(H_{0}\right)}\right)\right]=W_{R}$$

is the recharging work.

### 4.4. Extracting Process

The extracting process also fluctuates when we measure the battery’s energy in the charged state and the discharged state. We call κ the stochastic trajectory in the ergotropy extracting process and $\varpi_{\kappa }$ the stochastic extracted energy. The probability $p_{\kappa }$ of $\kappa =(m^{\prime },n)$ is the product of the transition probability from $|m^{\prime }\rangle$ to |n〉 under the permutation *u*, $P_{m^{\prime }\to n}^{\mathrm{ext}}={\left|\langle n|u|m^{\prime }\rangle \right|}^{2}=\delta_{\pi_{n},m^{\prime }}$, with the initial probability $e^{-\beta E_{m^{\prime }}^{0}}/Z_{0}$; see Equation (11). The averaged extracted energy,

(25) $$\langle \varpi_{\kappa }\rangle =\sum_{\kappa }\varpi_{\kappa }p_{\kappa }=\sum_{m^{\prime },n}\left(E_{m^{\prime }}-E_{n}\right)P_{m^{\prime }\to n}^{\mathrm{ext}}\frac{e^{-\beta E_{m^{\prime }}^{0}}}{Z_{0}}=\sum_{n}\left(E_{\pi_{n}}-E_{n}\right)\frac{e^{-\beta E_{\pi_{n}}^{0}}}{Z_{0}}=W\left(\omega_{\beta }\left(H_{0}\right)\right)$$

is the ergotropy Equation (12).

Equations (23) and (25) show the cycle’s consistency, where two processes, recharging (γ) and extracting (κ), connect the same states, $\omega_{\beta }\left(H_{0}\right)$ and $\sigma_{\omega_{\beta }\left(H_{0}\right)}$.

### 4.5. Fluctuating Efficiency for the Cycle

In terms of Equations (24) and (25), we have the thermodynamic efficiency $\eta_{\mathrm{th}}=\frac{W}{W_{R}}=\frac{\langle \varpi_{\kappa }\rangle }{{\langle w_{\gamma }\rangle }^{\left(\infty \right)}}$.

As the thermodynamic efficiency is the ratio of the ergotropy over the recharging work, the fluctuating efficiency [21] should be the ratio of their fluctuating equivalents. The fluctuating extracted energy is $\varpi_{\kappa }=E_{m^{\prime }}-E_{n}$, and the fluctuating work is $w_{\gamma }=E_{m}-E_{m}^{0}-\left(E_{n}-E_{n}^{0}\right)$. Therefore, we define the fluctuating efficiency as

(26) $$\eta_{\gamma \kappa }=\frac{\varpi_{\kappa }}{w_{\gamma }}=\frac{E_{m^{\prime }}-E_{n}}{E_{m}-E_{m}^{0}-\left(E_{n}-E_{n}^{0}\right)}.$$

Given the extracting trajectory κ, the probability of the recharging trajectory γ is $P_{m^{\prime }\to n}^{\mathrm{ext}}P_{n\to m}^{\infty }$. Thus, the joint probability for the processes κ and γ is

$$p_{\gamma \kappa }=\frac{e^{-\beta E_{m^{\prime }}^{0}}}{Z_{0}}P_{m^{\prime }\to n}^{\mathrm{ext}}P_{n\to m}^{\infty }=\frac{e^{-\beta E_{m^{\prime }}^{0}}}{Z_{0}}\delta_{\pi_{n},m^{\prime }}\frac{e^{-\beta E_{m}^{0}}}{Z_{0}},$$

and the distribution of the fluctuating efficiency is

(27) $$p_{\eta }\left(x\right)=\sum_{\gamma ,\kappa }\delta \left(x-\eta_{\gamma \kappa }\right)p_{\gamma \kappa }=\sum_{n,m}\delta \left(x-\frac{E_{\pi_{n}}-E_{n}}{E_{m}-E_{m}^{0}-\left(E_{n}-E_{n}^{0}\right)}\right)\frac{e^{-\beta (E_{m}^{0}+E_{\pi_{n}}^{0})}}{Z_{0}^{2}}.$$

To simplify the notation, we write this as

(28) $$p_{\eta }\left(x\right)=\sum_{n,m}\delta \left(x-\eta_{nm}\right)P_{n\to m},$$

with

(29) $$\eta_{nm}=\frac{E_{\pi_{n}}-E_{n}}{E_{m}-E_{m}^{0}-\left(E_{n}-E_{n}^{0}\right)},\;\mathrm{and}\;P_{n\to m}=\frac{e^{-\beta (E_{m}^{0}+E_{\pi_{n}}^{0})}}{Z_{0}^{2}}.$$

The probability $P_{n\to m}$ corresponds Equation (22), and we omit the superscript.

Trajectories with $w_{\gamma }=0$ and $\varpi_{\kappa }\neq 0$ have $|\eta_{\gamma \kappa }|=\infty$. Therefore, the average $\langle \eta_{\gamma \kappa }\rangle$ does not always exist, and if it does, $\eta_{\mathrm{th}}\neq \langle \eta_{\gamma \kappa }\rangle$, unless the stochastic work and efficiency are uncorrelated. In fact, $\langle \eta_{\gamma \kappa }w_{\gamma }\rangle =\langle \varpi_{\kappa }\rangle =W$. So only if $\langle \eta_{\gamma \kappa }w_{\gamma }\rangle =\langle \eta_{\gamma \kappa }\rangle W_{R}$ do we have $\langle \eta_{\gamma \kappa }\rangle =\eta_{\mathrm{th}}$. The thermodynamic and fluctuating efficiency can be very different.

The following section discusses efficiency fluctuations for the cycle, heat and work fluctuations for the recharging process and equilibrium fluctuations in two examples.

## 5. Examples

We illustrate our results in two simple examples. The first example is a single-qubit battery that we use to discuss equilibrium fluctuations (Section 4.2). The second example is a two-qubit battery where we compute heath and work distributions in a partial recharging process (Section 4.3). In both, we compute the fluctuating efficiency distribution (Section 4.5).

### 5.1. Single-Qubit Battery

An interesting protocol, with $H_{0}=-H_{S}$, was discussed in [26] for a system *S* interacting with systems *A*, which are copies of *S*. The corresponding process E has the remarkable equilibrium state

$$\omega_{\beta }\left(-H_{S}\right)=\sum_{n=1}^{N}\frac{e^{\beta E_{n}}}{Z_{+}}|n\rangle \langle n|,$$

with $Z_{+}=\mathrm{Tr}\left[e^{+\beta H_{S}}\right]$ between a system in the state $\omega_{\beta }\left(-H_{S}\right)$ with copies of itself in the Gibbs state $\omega_{\beta }\left(H_{S}\right)$.

In this subsection, we consider the battery *S* and auxiliary systems *A* identical qubits; i.e., the battery Hamiltonian is $H_{S}=\left(h/2\right)\sigma_{S}^{z}$, and the baths Hamiltonians are $H_{A}=\left(h/2\right)\sigma_{A}^{z}$, with h>0. Hereafter, $\sigma^{x},\sigma^{y}$ and $\sigma^{z}$ are Pauli matrices.

The coupling between the system and the bath qubit is

$$V=a(\sigma_{S}^{+}\sigma_{A}^{+}+\sigma_{S}^{-}\sigma_{A}^{-}),$$

with $\sigma^{\pm }=\left(\sigma^{x}\pm \sigma^{y}\right)/2$, and is such that $[\sigma_{A}^{z}-\sigma_{S}^{z},V]=0$, i.e., $H_{0}=-H_{S}$.

In the basis defined by $\sigma^{z}|↑\rangle =|↑\rangle$ and $\sigma^{z}|↓\rangle =-|↓\rangle$, the eigenvalues and eigenvectors of $H_{S}$ and $H_{0}$ are

(30) $$\begin{array}{cccccc} E_{2} & =h/2, & E_{2}^{0} & =-h/2, & |2\rangle & =|↑\rangle \end{array}$$

(31) $$\begin{array}{cccccc} E_{1} & =-h/2, & E_{1}^{0} & =h/2, & |1\rangle & =|↓\rangle \end{array}$$

and the ordering permutation is $\left(\pi_{1},\pi_{2}\right)=\left(2,1\right)$. Thus, on the above basis, the equilibrium state is

$$\omega_{\beta }\left(H_{0}\right)=\omega_{\beta }\left(-H_{S}\right)=\frac{e^{\beta \frac{h}{2}}}{Z}|2\rangle \langle 2|+\frac{e^{-\beta \frac{h}{2}}}{Z}|1\rangle \langle 1|,$$

and the passive state for the system is

$$\sigma_{\omega_{\beta }\left(H_{0}\right)}=\omega_{\beta }\left(H_{S}\right)=\frac{e^{-\beta \frac{h}{2}}}{Z}|2\rangle \langle 2|+\frac{e^{\beta \frac{h}{2}}}{Z}|1\rangle \langle 1|,$$

where $Z=Z_{+}=2\cosh\left(\beta h/2\right).$ With Equations (30) and (31), and the permutation π, we can evaluate the transition probabilities in Equation (29). The ergotropy of the battery in the equilibrium state $\omega_{\beta }\left(-H_{S}\right)$ is W=htanhβh/2. From Equations (23) and (24), we see that the thermodynamic efficiency of the process is $\eta_{\mathrm{th}}=1/2,$ independent of the inverse temperature β.

The recharging process in this single-qubit battery (1Q) is determined by the stochastic matrix (see Equation (21))

(32) $$T_{1Q}=\left(\begin{array}{cc} 1-\frac{e^{\beta \frac{h}{2}}}{Z}g\left(a,h\right) & \frac{e^{-\beta \frac{h}{2}}}{Z}g\left(a,h\right)\\ \frac{e^{\beta \frac{h}{2}}}{Z}g\left(a,h\right) & 1-\frac{e^{-\beta \frac{h}{2}}}{Z}g\left(a,h\right) \end{array}\right)$$

where $g\left(a,h\right)=\frac{a^{2}\sin^{2}\left(\tau \sqrt{h^{2}+a^{2}}/\hbar \right)}{h^{2}+a^{2}}$ and $Z=e^{\beta \frac{h}{2}}+e^{-\beta \frac{h}{2}}$. It is a regular stochastic matrix if g(a,h)≠0.

#### 5.1.1. Fluctuating Efficiency

The fluctuating efficiency (see Equation (29)) takes the values

$$\eta_{11}=-\eta_{22}=\infty ,\;\eta_{12}=\eta_{21}=\frac{1}{2}$$

Its distribution Equation (28) is

$$p_{\eta }\left(x\right)=\delta \left(x-\infty \right)P_{\infty }+\delta \left(x+\infty \right)P_{-\infty }+\delta \left(x-\frac{1}{2}\right)P_{\frac{1}{2}}$$

with

(33) $$P_{\infty }=P_{1\to 1}=P_{-\infty }=P_{2\to 2}=\frac{1}{Z^{2}},\;P_{\frac{1}{2}}=P_{1\to 2}+P_{2\to 1}=\frac{e^{\beta h}+e^{-\beta h}}{Z^{2}}$$

The explicit formulas at the right follow from Equation (29), which is valid if g(a,h)≠0 in $T_{1Q}$.

**Figure 1:**
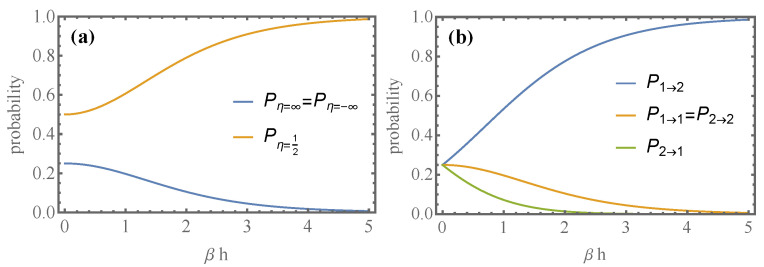
For the 1-qubit battery (**a**) Plots of $P_{\eta }$ (Equation (33)) as a function of βh. (**b**) Plots of $P_{1\to 2}=e^{\beta h}/Z^{2}$, $P_{2\to 1}=e^{-\beta h}/Z^{2}$ and $P_{1\to 1}=P_{2\to 2}=1/Z^{2}$ for the single-qubit battery (see Equation (29)). The charging process becomes deterministic as the temperature decreases, and the fluctuating efficiency equals the thermodynamic efficiency 1/2 with probability one.

In Figure 1a, we depict the probabilities $P_{\eta }$ as functions of βh and see that for βh≫1 with probability 1; the fluctuating efficiency equals the thermodynamic efficiency 1/2, because, as we see in Figure 1b, $P_{1\to 2}\to 1$, reflecting the charging character of the process.

**Figure 2:**
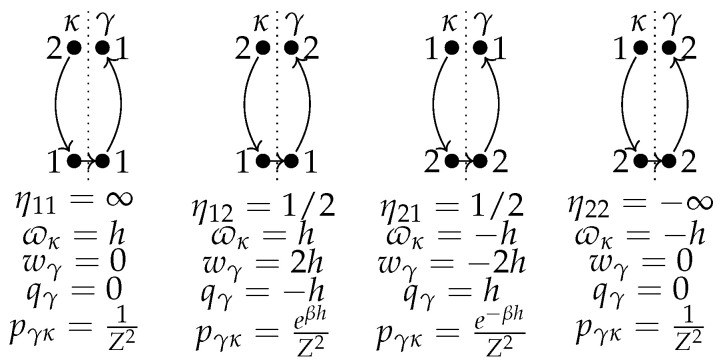
Diagrammatic representation of the κ and γ paths for the discharging-charging cycle in the single-qubit battery. Underneath each diagram, the associated value of the efficiency, extracted energy, work, heat, and probability are given.

The diagrams in Figure 2 depict κ transitions (left, up to down), followed by γ transitions (right, down to up). The values of all variables and their probability are given underneath.

The numbers correspond to the energy levels 1 and 2. In the limit of large temperature, β→0, all these processes have the same probability 1/4, while at low temperature β→∞, the probability of the second process goes to one and the others to zero. Only the third diagram has a transition assisted by heat, $q_{\gamma }=h$. We extract energy $w_{\gamma }=-2h<0$ in the γ process and invest $\varpi_{\kappa }=-h$ in the κ process. This cycle is the least likely. Its probability is $e^{-\beta h}/Z^{2}$ and decreases quickly as βh increases.

#### 5.1.2. Equilibrium Fluctuation

Let us analyze the fluctuations when maintaining the charged state, i.e., those of the process $\omega_{\beta }\left(H_{0}\right)\to E^{L}\left(\omega_{\beta }\left(H_{0}\right)\right)=\omega_{\beta }\left(H_{0}\right)$; see Section 4.2. As we can verify in the examples above, and as shown in [72], the transition matrices *T* for maps with equilibrium satisfy the detailed balance condition $T_{m|n}e^{-\beta E_{n}^{0}}=T_{n|m}e^{-\beta E_{m}^{0}}$. From this fact, it is simple to show that $P_{n\to m}^{\left(L\right)}=P_{m\to n}^{\left(L\right)}$ with $p_{\mathrm{ini}}\left(n\right)=e^{-\beta E_{n}^{0}}/Z_{0}$ in Equation (21).

We are interested in distinguishing fluctuations in an active equilibrium state from fluctuations in a Gibbs equilibrium state. The main difference is that the probability distribution of equilibrium work fluctuation is $p_{w}\left(x\right)\neq \delta \left(x\right)$ for the former, reflecting an active agent, and $p_{w}\left(x\right)=\delta \left(x\right)$ for the latter, reflecting a passive agent.

To investigate other differences, we consider our charging map E and a thermal map $E^{\mathrm{Thm}}$ for a qubit. The map $E^{\mathrm{Thm}}$ is obtained by coupling the qubit to an auxiliary thermal qubit with $V=a(\sigma_{S}^{+}\sigma_{A}^{-}+\sigma_{S}^{-}\sigma_{A}^{+})$ and tracing out the auxiliary system. The resulting map is thermal (i.e., a map with the Gibbs equilibrium state), and the transition matrix for this process is

$$T^{\mathrm{Thm}}=\left(\begin{array}{cc} 1-\frac{e^{-\beta \frac{h}{2}}}{Z}g\left(a,0\right) & \frac{e^{\beta \frac{h}{2}}}{Z}g\left(a,0\right)\\ \frac{e^{-\beta \frac{h}{2}}}{Z}g\left(a,0\right) & 1-\frac{e^{\beta \frac{h}{2}}}{Z}g\left(a,0\right) \end{array}\right)$$

where $g\left(a,0\right)=\sin^{2}\left(\tau a/\hbar \right)$ and $Z=e^{\beta \frac{h}{2}}+e^{-\beta \frac{h}{2}}.$ $T^{\mathrm{Thm}}$ is a regular stochastic matrix if g(a,0)≠0. The most crucial difference between $T^{\mathrm{Thm}}$ and $T_{1Q}$ in Equation (32) is the position of the factors $e^{\pm \beta h/2}$.

For the charging map, one can show $P_{2\to 2}^{\left(L\right)}>P_{1\to 1}^{\left(L\right)}$, reflecting the higher population of the excited state in the active equilibrium. Instead, for the thermal map, $P_{1\to 1}^{(L)\mathrm{Thm}}>P_{2\to 2}^{(L)\mathrm{Thm}}$, reflecting the higher population of the ground state in Gibbs equilibrium. On the other hand, energy fluctuations due to 1↔2 transitions are qualitatively similar if g(a,h)≈g(a,0) for processes with finite *L* but are indistinguishable for L→∞. Indeed, for L→∞, we have

$$P_{1\to 2}^{(\infty )\mathrm{Thm}}=P_{2\to 1}^{(\infty )\mathrm{Thm}}=\frac{1}{Z^{2}},\;P_{1\to 1}^{(\infty )\mathrm{Thm}}=\frac{e^{\beta h}}{Z^{2}},\;P_{2\to 2}^{(\infty )\mathrm{Thm}}=\frac{e^{-\beta h}}{Z^{2}}$$

and for the charging map,

$$P_{2\to 1}^{\left(\infty \right)}=P_{1\to 2}^{\left(\infty \right)}=\frac{1}{Z^{2}},\;P_{2\to 2}^{\left(\infty \right)}=\frac{e^{\beta h}}{Z^{2}},\;P_{1\to 1}^{\left(\infty \right)}=\frac{e^{-\beta h}}{Z^{2}}.$$

Thus, these processes are very similar at the level of energy fluctuations.

### 5.2. Two-Qubit Battery

We consider a two-qubit battery with Hamiltonian [26]

$$H_{S}=\frac{h}{2}\left(\sigma_{1}^{z}+\sigma_{2}^{z}\right)+J\left(\sigma_{1}^{x}\sigma_{2}^{x}+\sigma_{1}^{y}\sigma_{2}^{y}\right),$$

coupled with

$$V=J^{\prime }\left(\sigma_{A}^{x}\sigma_{1}^{x}+\sigma_{A}^{y}\sigma_{1}^{y}\right),$$

to auxiliary systems with Hamiltonian $H_{A}=\frac{h}{2}\sigma_{A}^{z}$ in the thermal state. The corresponding map E has the equilibrium state $\omega_{\beta }\left(H_{0}\right)$ with $H_{0}=\frac{h}{2}\left(\sigma_{1}^{z}+\sigma_{2}^{z}\right)$.

The eigenvalues and eigenvectors of $H_{S}$ and $H_{0}$ in the basis defined by $\sigma^{z}|↑\rangle =|↑\rangle$ and $\sigma^{z}|↓\rangle =-|↓\rangle$ are

(34) $$\begin{array}{cccccc} E_{3} & =h, & E_{3}^{0} & =h, & |3\rangle & =|↑↑\rangle , \end{array}$$

(35) $$\begin{array}{cccccc} E_{4} & =2J, & E_{4}^{0} & =0, & |4\rangle & =\left(|↑↓\rangle +|↓↑\rangle \right)/\sqrt{2}, \end{array}$$

(36) $$\begin{array}{cccccc} E_{1} & =-2J, & E_{1}^{0} & =0, & |1\rangle & =\left(|↑↓\rangle -|↓↑\rangle \right)/\sqrt{2}, \end{array}$$

(37) $$\begin{array}{cccccc} E_{2} & =-h, & E_{2}^{0} & =-h, & |2\rangle & =|↓↓\rangle . \end{array}$$

We take 2J>h>0 such that $E_{i+1}>E_{i}$. The permutation that orders $E_{\pi_{i+1}}^{0}\geq E_{\pi_{i}}^{0}$ is $\left(\pi_{1},\pi_{2},\pi_{3},\pi_{4}\right)=\left(2,1,4,3\right)$. Thus, on the above basis, the equilibrium state is

$$\omega_{\beta }\left(H_{0}\right)=\frac{e^{-\beta h}}{Z_{0}}|3\rangle \langle 3|+\frac{1}{Z_{0}}\left(|1\rangle \langle 1|+|4\rangle \langle 4|\right)+\frac{e^{\beta h}}{Z_{0}}|2\rangle \langle 2|,$$

and the passive state for the system is

$$\sigma_{\omega_{\beta }\left(H_{0}\right)}=\frac{e^{\beta h}}{Z_{0}}|1\rangle \langle 1|+\frac{1}{Z_{0}}\left(|2\rangle \langle 2|+|3\rangle \langle 3|\right)+\frac{e^{-\beta h}}{Z_{0}}|4\rangle \langle 4|,$$

where $Z_{0}=2+2\cosh\left(\beta h\right).$ The ergotropy of the equilibrium state $W=\mathrm{Tr}[H_{S}\left(\omega_{\beta }\left(H_{0}\right)-\sigma_{\omega_{\beta }\left(H_{0}\right)}\right)]$ is

$$W=\left(2J-h\right)\frac{\sinh\beta h}{1+\cosh\beta h}.$$

The work performed in the charging process $\sigma_{\omega_{\beta }\left(H_{0}\right)}\to \omega_{\beta }\left(H_{0}\right)$ is

$$W_{R}=2J\frac{\sinh\beta h}{1+\cosh\beta h}.$$

We see that the thermodynamic efficiency is $\eta_{\mathrm{th}}=W/W_{R}=1-\frac{h}{2J}$ independently of the inverse temperature β.

The recharging process in this two-qubit battery (2Q) is determined by the stochastic matrix (see Equation (21))

(38) $$T_{2Q}=\frac{1}{{\left(J^{2}+J^{\prime 2}\right)}^{2}}\left(\begin{array}{cccc} \Phi^{2} & \frac{2}{\left(1+e^{\beta h}\right)}\Phi \Psi & \frac{2e^{\beta h}}{\left(1+e^{\beta h}\right)}\Phi \Psi & \Psi^{2}\\ \frac{2e^{\beta h}}{\left(1+e^{\beta h}\right)}\Phi \Psi & \frac{e^{\beta h}{\left(J^{2}+J^{\prime 2}\right)}^{2}+\Delta }{\left(1+e^{\beta h}\right)} & 0 & \frac{2e^{\beta h}}{\left(1+e^{\beta h}\right)}\Phi \Psi \\ \frac{2}{\left(1+e^{\beta h}\right)}\Phi \Psi & 0 & \frac{{\left(J^{2}+J^{\prime 2}\right)}^{2}+e^{\beta h}\Delta }{\left(1+e^{\beta h}\right)} & \frac{2}{\left(1+e^{\beta h}\right)}\Phi \Psi \\ \Psi^{2} & \frac{2}{\left(1+e^{\beta h}\right)}\Phi \Psi & \frac{2e^{\beta h}}{\left(1+e^{\beta h}\right)}\Phi \Psi & \Phi^{2} \end{array}\right),$$

with

$$\Phi =J^{2}+J^{\prime 2}\cos^{2}\left(\frac{\tau }{\hbar }\sqrt{J^{2}+J^{\prime 2}}\right),\;\Psi =J^{\prime 2}\sin^{2}\left(\frac{\tau }{\hbar }\sqrt{J^{2}+J^{\prime 2}}\right),\;\Delta ={\left(\Phi -\Psi \right)}^{2},$$

which is a regular stochastic matrix excepts at points with Ψ=0 or Φ=0, as one can check by computing $T^{2}$.

#### 5.2.1. Fluctuating Efficiency

For the fluctuating efficiency Equation (29), we have

(39) $$\begin{array}{cc} \eta_{12} & =\eta_{13}=\eta_{21}=\eta_{34}=\eta_{42}=\eta_{43}=1-\frac{h}{2J} \end{array}$$

(40) $$\begin{array}{cc} \eta_{14} & =\eta_{41}=\frac{1}{2}\left(1-\frac{h}{2J}\right) \end{array}$$

(41) $$\begin{array}{cc} \eta_{32} & =-\eta_{23}=\infty \end{array}$$

(42) $$\begin{array}{cc} \eta_{24} & =\eta_{31}=-\left(1-\frac{h}{2J}\right) \end{array}$$

and $\eta_{11}=\eta_{33}=-\eta_{22}=-\eta_{44}=\infty$. Its distribution follows from Equation (28), and it is

$$\begin{array}{c} p_{\eta }\left(x\right)=\delta \left(x-\infty \right)P_{\infty }+\delta \left(x+\infty \right)P_{-\infty }+\delta \left(x-1+\frac{h}{2J}\right)P_{\left(1-\frac{h}{2J}\right)}+\\ \delta \left(x+1-\frac{h}{2J}\right)P_{-(1-\frac{h}{2J})}+\delta \left(x-\frac{1}{2}+\frac{h}{4J}\right)P_{\left(1/2\right)\left(1-\frac{h}{2J}\right)}\; \end{array}$$

with

(43) $$\begin{array}{cc} \; & P_{\infty }=P_{3\to 2}+P_{1\to 1}+P_{3\to 3}=\frac{2e^{\beta h}+e^{-\beta h}}{Z_{0}^{2}}, \end{array}$$

(44) $$\begin{array}{cc} \; & P_{-\infty }=P_{2\to 3}+P_{2\to 2}+P_{4\to 4}=\frac{e^{\beta h}+2e^{-\beta h}}{Z_{0}^{2}}, \end{array}$$

(45) $$\begin{array}{cc} \; & P_{\left(1-\frac{h}{2J}\right)}=P_{1\to 2}+P_{1\to 3}+P_{2\to 1}+P_{3\to 4}+P_{4\to 2}+P_{4\to 3}=\frac{2+{\left(e^{\beta h}+e^{-\beta h}\right)}^{2}}{Z_{0}^{2}}, \end{array}$$

(46) $$\begin{array}{cc} \; & P_{-(1-\frac{h}{2J})}=P_{3\to 1}+P_{2\to 4}=\frac{2}{Z_{0}^{2}}, \end{array}$$

(47) $$\begin{array}{cc} \; & P_{\left(1/2\right)\left(1-\frac{h}{2J}\right)}=P_{1\to 4}+P_{4\to 1}=\frac{\left(e^{-\beta h}+e^{\beta h}\right)}{Z_{0}^{2}}. \end{array}$$

The explicit formulas on the right follow from Equation (29) and are valid for parameters τ,J and $J^{\prime }$ in which $T_{2Q}$ is regular.

**Figure 3:**
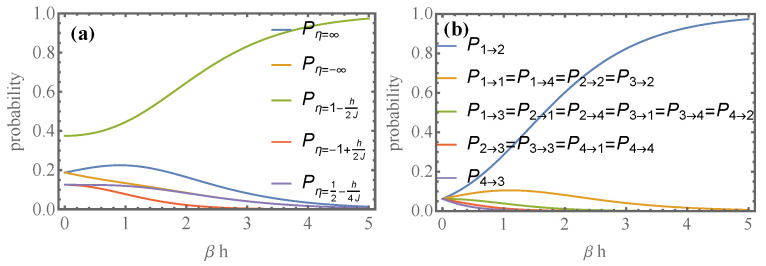
For the 2-qubit battery: (**a**) plots of $P_{\eta }$ as a function of βh and (**b**) plots of $P_{n\to m}$ given by Equation (29) for the two-qubit battery.We observe that as temperature decreases, the 1→2 transition dominates. Fluctuations become negligible, and the fluctuating efficiency equals the thermodynamic efficiency with a probability of one.

In Figure 3a, we plot the probabilities $P_{\eta }$ in Equations (44)–(47) as a function of βh. We see that for small βh, the average efficiency does not exist. On the other hand, when βh≫1, the efficiency goes to the thermodynamic efficiency with a probability of one because the work becomes deterministic.

**Figure 4:**
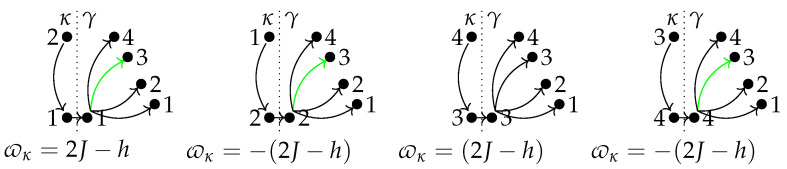
Diagrammatic representation of the κ and γ paths for the discharging-charging cycle in the two-qubit battery. Underneath each diagram, the associated value of the extracted energy is given.

The diagrams in Figure 4, summarize all possible extracting–recharging cycles. The numbers correspond to the energy levels 1,2,3, and 4. Since extracting the ergotropy only allows transitions $\kappa :m^{\prime }\to n$ with $m^{\prime }=\pi_{n}$, we have four possible processes κ. From Figure 3b, we see that the only process with a high probability for large βh is the sequence $2\overset{\kappa }{\to }1\overset{\gamma }{\to }2$ contained in the first diagram. Its efficiency equals thermodynamic efficiency. Green arrows are processes assisted by heat ($q_{\gamma }>0$). These have very low probabilities, as depicted in Figure 3b. In Figure 3b, we see that $P_{1\to 2}$ goes to one in that limit. Second in importance are $P_{1\to 4}$, associated with the largest charge, but in the extracting κ process, one has $4\overset{\kappa }{\to }3$, and the γ process starting in 3 reaches 1,2, or 4 with similar probabilities and the battery is noisy.

#### 5.2.2. Heat and Work Fluctuations in the Partial Recharging Process

Here, we consider the process $E^{L}$ starting in the state $\sigma_{\omega_{\beta }\left(H_{0}\right)}$ and evaluate the heat and work distributions. Hence, we consider Equations (19) and (20) with $P_{n\to m}^{\left(L\right)}={\left(T^{L}\right)}_{m|n}\frac{e^{-\beta E_{\pi_{n}}^{0}}}{Z0}$, with the permutation π ordering the eigenvalues of $H_{0}$ by increasing values.

For the two-qubit battery, we obtain

(48) $$\begin{array}{cc} p_{w}^{\left(L\right)}\left(x\right) & =\delta \left(x\right)A_{0}^{\left(L\right)}+\delta \left(x-4J\right)A_{1}^{\left(L\right)}+\delta \left(x-2J\right)A_{3}^{\left(L\right)}+\delta \left(x+4J\right)A_{2}^{\left(L\right)}+\delta \left(x+2J\right)A_{4}^{\left(L\right)}, \end{array}$$

(49) $$\begin{array}{cc} p_{q}^{\left(L\right)}\left(x\right) & =\delta \left(x\right)B_{0}^{\left(L\right)}+\delta \left(x-h\right)B_{4}^{\left(L\right)}+\delta \left(x-2h\right)B_{2}^{\left(L\right)}+\delta \left(x+h\right)B_{3}^{\left(L\right)}+\delta \left(x+2h\right)B_{1}^{\left(L\right)} \end{array}$$

with

(50) $$\begin{array}{cccc} A_{0}^{\left(L\right)} & =P_{2\to 3}^{\left(L\right)}+P_{3\to 2}^{\left(L\right)}, & B_{0}^{\left(L\right)} & =P_{1\to 4}^{\left(L\right)}+P_{4\to 1}^{\left(L\right)}, \end{array}$$

(51) $$\begin{array}{cccc} A_{1}^{\left(L\right)} & =P_{1\to 4}^{\left(L\right)}, & B_{1}^{\left(L\right)} & =P_{3\to 2}^{\left(L\right)}, \end{array}$$

(52) $$\begin{array}{cccc} A_{2}^{\left(L\right)} & =P_{4\to 1}^{\left(L\right)}, & B_{2}^{\left(L\right)} & =P_{2\to 3}^{\left(L\right)}, \end{array}$$

(53) $$\begin{array}{cccc} A_{3}^{\left(L\right)} & =P_{1\to 2}^{\left(L\right)}+P_{1\to 3}^{\left(L\right)}+P_{2\to 4}^{\left(L\right)}+P_{3\to 4}^{\left(L\right)}, & B_{3}^{\left(L\right)} & =P_{1\to 2}^{\left(L\right)}+P_{3\to 1}^{\left(L\right)}+P_{4\to 2}^{\left(L\right)}+P_{3\to 4}^{\left(L\right)}, \end{array}$$

(54) $$\begin{array}{cccc} A_{4}^{\left(L\right)} & =P_{2\to 1}^{\left(L\right)}+P_{3\to 1}^{\left(L\right)}+P_{4\to 2}^{\left(L\right)}+P_{4\to 3}^{\left(L\right)}, & B_{4}^{\left(L\right)} & =P_{2\to 1}^{\left(L\right)}+P_{1\to 3}^{\left(L\right)}+P_{2\to 4}^{\left(L\right)}+P_{4\to 3}^{\left(L\right)}, \end{array}$$

where $A_{i}^{\left(L\right)}\neq B_{i}^{\left(L\right)}$ for finite *L* but $A_{i}^{\left(\infty \right)}=B_{i}^{\left(\infty \right)}$ with

$$A_{0}^{\left(\infty \right)}=\frac{6\cosh\beta h}{Z_{0}^{2}},\;A_{1}^{\left(\infty \right)}=\frac{e^{\beta h}}{Z_{0}^{2}},\;A_{2}^{\left(\infty \right)}=\frac{e^{-\beta h}}{Z_{0}^{2}},\;A_{3}^{\left(\infty \right)}=\frac{e^{2\beta h}+3}{Z_{0}^{2}},\;A_{4}^{\left(\infty \right)}=\frac{3+e^{-2\beta h}}{Z_{0}^{2}}.$$

This means that the average work $W^{\left(L\right)}$ and average heat $Q^{\left(L\right)}$

$$W^{\left(L\right)}=2J\left(A_{3}^{\left(L\right)}-A_{4}^{\left(L\right)}\right)+4J\left(A_{1}^{\left(L\right)}-A_{2}^{\left(L\right)}\right)\underset{L\to \infty }{\to }\frac{2J\sinh\beta h}{1+\cosh\beta h}$$

$$Q^{\left(L\right)}=h\left(B_{4}^{\left(L\right)}-B_{3}^{\left(L\right)}\right)+2h\left(B_{2}^{\left(L\right)}-B_{1}^{\left(L\right)}\right)\underset{L\to \infty }{\to }\frac{-h\sinh\beta h}{1+\cosh\beta h}$$

become proportional when L→∞.

**Figure 5:**
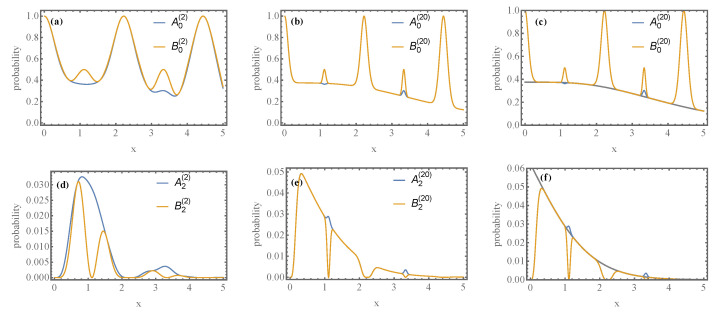
Plots of the probabilities $A_{i}^{\left(L\right)}$ and $B_{i}^{\left(L\right)}$, with L=2 at the left (**a**,**d**) and L=20 at the center (**b**,**e**) with i=0 at the top (**a**,**b**) and i=2 at the bottom (**d**,**e**). On the right (**c**,**f**), we superpose the analytical result $A_{i}^{\left(\infty \right)}$ and $B_{i}^{\left(\infty \right)}$ to the data at the center for L=20. For the numerical computation, we take $\beta =\tau /\hbar =1,J=J^{\prime }=x$ and h=0.6x. We observe that besides neighborhoods of points where $T_{2Q}$ is not regular, the theoretical prediction in Equation (22) is observed after L≈20 iterations.

Since Markov chains converge exponentially quickly to the stationary state, it is unnecessary to consider a large *L* to observe the asymptotic distribution. However, since the convergence rate depends on the map’s parameters, we see deviations from it near the points where Φ=0 or Ψ=0 in Equation (38). To illustrate this point, we plot in Figure 5 the probabilities $A_{0}^{\left(L\right)},B_{0}^{\left(L\right)},A_{2}^{\left(L\right)}$, and $B_{2}^{\left(L\right)}$ for various values of *L* and varying map parameters.

Figure 5 shows that for L=20, convergence is achieved with a duration of each iteration τ/ℏ=1. Note that the dependence on τ in $T_{2Q}$ is periodic; see Equation (38). In the limit L→∞,τ→0 and $J^{\prime }=j/\sqrt{\tau }$, the dynamics of the battery has the Lindblad form [69] and converges exponentially quickly to the equilibrium distribution.

## 6. Discussion

We have studied stochastic fluctuations in repeated interaction processes subjected to the two-point energy-measurement scheme. Because map E has an equilibrium state, all quantities are expressed in terms of system properties simplifying their study because one does not require measuring the environment. We have shown that the equilibrium distribution of the map dominates the distributions, except at particular points in the parameter space of the map, where its details become essential. Near these zones, the convergence rate towards the asymptotic value is low, requiring larger values of *L* to reach it. The quantum aspect of the system is relevant near these zones since the Planck constant appears in the parameters that set the convergence rate to the stationary state. We have applied these results to study active equilibrium fluctuations, fluctuations in the charging process of a quantum battery, and efficiency fluctuations of the cycle charging and extracting energy for the battery in two examples. The fluctuating efficiency converges to the thermodynamic efficiency of these examples in the low-temperature limit, where the batteries operate in the cycle $2\overset{\kappa }{\to }1\overset{\gamma }{\to }2$ and are reliable. On the other hand, at large temperatures, where heat assists some transitions, all cycles are probable, and the battery is unreliable.

For future research, it would be interesting to extend the results obtained here for single-cycle efficiency to the case of an arbitrary number of cycles. As this number increases, universal statistical behaviors have been shown to appear in other machines [58,59,68]. Likewise, considering the collective boost in power for dissipative quantum batteries [79] and the result in [43], studying fluctuations as the number of batteries increases is of similar interest.

## Data Availability

Not applicable.

## Appendix A. Distributions for Maps with Equilibrium

Let us justify Equations (15) and (20). Equations (18) and (19) follow from the same argument.

We can consider that the system *S* and all the copies of system *A* start as uncorrelated in a product state. We measure the energy of that system and project the state to $|i_{1}\cdots i_{L}n\rangle$ with a probability $\frac{e^{-\beta \sum_{k=1}^{L}\varepsilon_{i_{k}}}}{Z_{A}^{L}}p_{\mathrm{ini}}\left(n\right)$ because the copies of *A* are in the Gibbs state. Then, the full system evolves unitarily by composing the unitary evolution, where at each time, only the system *S* with a copy *i* of *A* is interacting. This is represented by the product $U_{L}\cdots U_{1}$, and the global state is $U_{L}\cdots U_{1}|i_{1}\cdots i_{L}n\rangle$. Then, we measure the energy of *S* and of each copy of *A*. According to the Born rule, after the measurement, the total system is the state $|j_{1}\cdots j_{L}m\rangle$ with a probability of

(A1) $$P_{\gamma }^{\left(L\right)}=|\langle j_{1}\cdots j_{L}n_{a_{L}}|U_{L}\cdots U_{1}|i_{1}\cdots i_{L}n_{a_{0}}{\rangle |}^{2}\frac{e^{-\beta \sum_{n=1}^{L}\varepsilon_{i_{n}}}}{Z_{b}^{L}}p_{i}\left(n_{a_{0}}\right).$$

More details can be found in [72].

We use these results to derive Equation (20), and by extension, all other distributions for maps with equilibrium. Consider that

$$\langle j_{1}\cdots j_{L}n_{a_{L}}|U_{L}\cdots U_{1}|i_{1}\cdots i_{L}n_{a_{0}}\rangle =\sum_{a_{1}a_{2}..a_{L-1}}\langle n_{a_{L}}j_{L}|U_{L}|n_{a_{L-1}}i_{L}\rangle \cdots \langle n_{a_{2}}j_{2}|U_{2}|n_{a_{1}}i_{2}\rangle \langle n_{a_{1}}j_{1}|U_{1}|n_{a_{0}}i_{1}\rangle$$

Because $[H_{0}+H_{A},U_{k}]=0$, the generic transition $\langle n_{a_{k}}j_{k}|U_{k}|n_{a_{k-1}}i_{k}\rangle =0$ unless $E_{a_{k}}^{0}+\varepsilon_{j_{k}}=E_{a_{k-1}}^{0}+\varepsilon_{i_{k}}$. Thus, in every trajectory γ with non-vanishing probability, we have

$$q_{\gamma }=\sum_{k}\left(\varepsilon_{i_{k}}-\varepsilon_{j_{k}}\right)=\sum_{k}\left(E_{a_{k}}^{0}-E_{a_{k-1}}^{0}\right)=E_{a_{L}}^{0}-E_{a_{1}}^{0}.$$

Hence

$$p_{q}^{\left(L\right)}\left(x\right)=\sum_{\gamma }\delta \left(x-q_{\gamma }\right)P_{\gamma }^{\left(L\right)}=\sum_{\gamma }\delta \left(q-\left(E_{a_{L}}^{0}-E_{a_{0}}^{0}\right)\right)P_{\gamma }^{\left(L\right)}=\sum_{a_{L},a_{0}}\delta \left(q-\left(E_{a_{L}}^{0}-E_{a_{0}}^{0}\right)\right)\sum_{\gamma :a_{L},a_{0}}P_{\gamma }^{\left(L\right)},$$

where in the last sum, we add over all trajectories γ starting at $n_{a_{0}}$ and ending at $n_{a_{L}}$. This corresponds to taking the traces over all systems *A* that interacted with *S* and thus $\sum_{\gamma :a_{L},a_{0}}P_{\gamma }^{\left(L\right)}=\langle n_{a_{L}}|E^{L}\left(|n_{a_{0}}\rangle \langle n_{a_{0}}|\right)|n_{a_{L}}\rangle p_{i}\left(n_{a_{0}}\right)$.
